# Educational intervention performed by nurses for blood pressure control: a systematic review with meta-analysis *

**DOI:** 10.1590/1518-8345.6648.3930

**Published:** 2023-05-12

**Authors:** Lariza Martins Falcão, Maria Vilaní Cavalcante Guedes, José Wicto Pereira Borges, Grazielle Roberta Freitas da Silva

**Affiliations:** 1 Universidade Estadual do Ceará, Fortaleza, CE, Brasil.; 2 Universidade Federal do Piauí, Teresina, PI, Brasil.

**Keywords:** Hypertension, Nursing, Health Education, Primary Health Care, Systematic Review, Meta-Analysis, Hipertensión, Enfermería, Educación para la Salud, Atención Primaria de Salud, Revisión Sistemática, Metaanálisis, Hipertensão, Enfermagem, Educação em Saúde, Atenção Primária à Saúde, Revisão Sistemática, Metanálise

## Abstract

**Objetivo:**

to assess the effect of an educational intervention performed by nurses for blood pressure control in people with arterial hypertension, when compared to usual care.

**Método:**

a systematic review with meta-analysis of randomized clinical trials, conducted in six databases. The studies included were those in which an educational intervention was performed by nurses on people with arterial hypertension. The risk of bias was assessed by means of the Risk of Bias Tool, the meta-analysis was performed in the Review Manager software and certainty of the evidence was calculated in the Grading of Recommendations Assessment, Development and Evaluation system.

**Resultados:**

a total of 1,692 studies were found, which were peer-reviewed, including eight of them in the meta-analysis. The meta-analysis was calculated for the “systolic blood pressure” and diastolic blood pressure” outcomes, in subgroups by time and by intervention performance type. For the in-person educational intervention, performed individually combined with a group activity, the effect estimate was -12.41 mmHg (95% Confidence Interval: from -16.91 to -7.91, p<0,00001) for systolic pressure and -5.40 mmHg (95% Confidence Interval: from -7.98 to -2.82, p<0,00001) for diastolic pressure, with high certainty of evidence.

**Conclusión:**

the educational intervention performed by nurses, individually and combined with a group activity, presents a statistically significant clinical effect. PROSPERO registration No.: CRD42021282707.

## Abstract (es)

**Objetivo:**

evaluar el efecto de la intervención educativa que realizan los enfermeros para controlar la presión arterial en personas con hipertensión arterial, en comparación con los cuidados habituales.

**Método:**

revisión sistemática con metaanálisis de ensayos clínicos aleatorizados realizada en seis bases de datos. Se incluyeron estudios en los cuales el enfermero llevó a cabo la intervención educativa en la persona con hipertensión arterial. El riesgo de sesgo se evaluó mediante la herramienta Risk of Bias Tool, el metaanálisis se hizo utilizando el software Review Manager y la certeza de la evidencia se calculó usando el sistema Grading of Recommendations Assessment, Development and Evaluation.

**Resultados:**

se encontraron 1692 estudios revisados por pares y se incluyeron ocho artículos en el metaanálisis. El metaanálisis se calculó para el resultado presión arterial sistólica y presión arterial diastólica, subagrupados por tiempo y tipo de implementación de la intervención. Para la intervención educativa presencial, realizada d forma individual combinada con actividad grupal, la estimativa del efecto fue -12,41 mmHg (Intervalo de Confianza 95%, -16,91 a -7,91, p<0,00001) para la presión sistólica y -5,40 mmHg (Intervalo 95% Confianza, -7,98 a -2,82, p<0,0001) para la presión diastólica, con certeza de evidencia alta.

**Conclusión:**

a intervención educativa realizada por el enfermero, de forma individual combinada con la actividad grupal, tiene efecto clínico y estadísticamente significativo. Registro PROSPERO: CRD42021282707.

## Abstract (pt)

**Objetivo:**

avaliar o efeito da intervenção educativa realizada por enfermeiros para controle da pressão arterial em pessoas com hipertensão arterial, comparada com cuidado habitual.

**Método:**

revisão sistemática com metanálise de ensaios clínicos randomizados realizada em seis bases de dados. Foram incluídos estudos em que a intervenção educativa foi realizada pelo enfermeiro à pessoa com hipertensão arterial. O risco de viés foi avaliado pela Risk of Bias Tool, a metanálise no software Review Manager e a certeza da evidência no sistema Grading of Recommendations Assessment, Development and Evaluation.

**Resultados:**

foram encontrados 1692 estudos, revisados por pares, e oito artigos foram incluídos na metanálise. A metanálise foi calculada para o desfecho pressão arterial sistólica e pressão arterial diastólica, em subgrupo por tempo e por tipo de execução da intervenção. Para a intervenção educativa presencial, realizada de modo individual combinada com atividade de grupo, a estimativa de efeito foi de -12.41 mmHg (Intervalo de Confiança 95%, -16.91 a -7.91, p<0.00001) para pressão arterial sistólica e -5.40 mmHg (Intervalo de Confiança 95%, -7.98 a -2.82, p<0.0001) para pressão arterial diastólica, com certeza da evidência alta.

**Conclusão:**

a intervenção educativa realizada pelo enfermeiro, de modo individual combinada com atividade de grupo, apresenta efeito clínico e estatisticamente significativo. Registro PROSPERO: CRD42021282707.

**Highlights:**

**(1)**The in-person educational intervention contributes in the treatment of hypertensive individuals.

**(2)**The in-person educational intervention collaborates in reducing blood pressure values.

**(3)**The in-person educational intervention performed by nurses is clinically effective.

## Introduction

Hypertension is recognized as one of the most important risk factors for all-cause mortality, in addition to being the leading cause of cardiovascular morbidity and mortality and disability worldwide. Thus, it becomes necessary to establish a care plan focused on three dimensions: therapeutic actions involving and not involving medications, educational actions and self-care ^( 1)^ . 

Maintaining the patient’s motivation to adhering to the treatment is perhaps one of the most arduous challenges faced by health professionals in relation to the care of people with arterial hypertension, especially in the Primary Health Care context, which is why it is always necessary to recognize the person’s will to undergo the treatment and understand their motivations and reasons for non-adherence to the therapy ^( 2- 3)^ . It is in the Primary Health Care environment that it is possible to develop comprehensive patient care, including health promotion and protection, in addition to being the gateway to the Unified Health System ( Sistema Único de Saúde, SUS) and the communication center for the entire Health Care Network to the SUS ^( 4)^ . 

In order to reach the goals that contribute to therapeutic adherence in people with arterial hypertension, and thus lead to improving their quality of health and life, nurses have been seeking support in interventions that collaborate for effective clinical Nursing care, which may contribute to achieving blood pressure control in people with arterial hypertension. Nursing interventions in Primary Health Care seek to enable an improvement in the patient’s clinical condition and to improve care promotion in order to contribute to health care and to the prevention of arterial hypertension cases ^( 5)^ . 

Educational interventions are prioritized among the ones that nurses can perform to care for people with arterial hypertension. According to the Nursing Interventions Classification (NIC), the health education intervention aims at developing and providing instructions and learning experiences to facilitate a voluntary behavioral adaptation that promotes the health of individuals, families, groups or communities ^( 6)^ . Among the educational strategies to be adopted by nurses, those that are carried out with longer monitoring periods, that perform interactions between small groups and that are based on the care and partnership model deserve attention ^( 7)^ . 

Educational interventions carried out for people with arterial hypertension should aim at reducing the blood pressure levels since, according to data from a meta-analysis performed in 2016, it was verified that a 10 mmHg reduction in systolic blood pressure significantly decreases by 20% the risk of major cardiovascular disease events (relative risk: 0.80, 95% confidence interval: 0.77-0.83) similarly across several population subgroups, regardless of high blood pressure onset ^( 8)^ . 

Therefore, in view of the diversity of educational interventions that can be carried out by nurses on people with arterial hypertension aiming at blood pressure control, it is pertinent to seek scientific evidence on the theme in question, justifying this review. In this perspective, the objective of this study is to assess the effect of an educational intervention performed by nurses for blood pressure control in people with arterial hypertension, when compared to usual care.

## Method

### Type of study

This is a systematic literature review with meta-analysis written according to the items proposed in the Preferred Reporting Items for Systematic Reviews and Meta-Analyses (PRISMA) ^( 9)^ as well as to the recommendations set forth in the Cochrane Handbook for Systematic Reviews of Interventions in Health ^( 10)^ . This type of study is a planned process, which summarizes diverse evidence from primary studies, with pre-specified eligibility criteria, to answer a specific research question and provide the best evidence about a given intervention ^( 10)^ . The protocol of this review was previously published in the International Prospective Register of Systematic Reviews (PROSPERO) platform. 

### Eligibility criteria

In order to define the eligibility criteria, the Population, Intervention, Control, Outcome and Study type (PICOS) strategy ^( 10)^ and the research question were considered. For this systematic review with meta-analysis, the PICOS mnemonic was used, where P (Population) refers to people with arterial hypertension, I (Intervention) to the educational intervention performed by nurses, C (Control) to usual care, O (Outcome) to systolic blood pressure (SBP) and diastolic blood pressure (DBP) values, and S (Study type) to randomized controlled clinical trials. Based on the aforementioned, the following research question was formulated: When compared to usual care, how effective are educational interventions performed by nurses in reducing blood pressure in people with arterial hypertension? 

The inclusion criteria to select the studies were as follows: people diagnosed with primary arterial hypertension according to the protocol or guideline followed in the study and aged ≥ 18 years old; educational intervention performed by nurses in person, face-to-face, on the patients, in addition to the Nursing consultation recommended in the health institution and usual care understood as the care provided to people with arterial hypertension according to the routine previously established by the health institution where the patient is regularly monitored by health professionals.

No restrictions were established regarding language and year of publication of the studies. The exclusion criteria for the studies were as follows: people with secondary arterial hypertension and pregnant women; educational intervention carried out by a multidisciplinary team or by another non-Nursing professional and when the Control Group received an intervention in addition to usual care.

### Information source

The search was conducted in the following databases: MEDLINE (Medical Literature Analysis and Retrieval System Online) via PubMed, LILACS ( Literatura Latino-Americana e do Caribe em Ciências da Saúde) via Biblioteca Virtual em Saúde (BVS), EMBASE (Excerpta Medica dataBase), CINAHL (Cumulative Index to Nursing and Allied Health Literature), Scopus and Web of Science. In addition, a manual search was conducted in the references of the studies selected. The articles were searched in May 2022. 

### Search strategy

Three controlled vocabularies in health were used for the search strategy, namely: Medical Subject Headings (MeSH) for MEDLINE, SCOPUS and Web of Science databases; Descriptors in Health Sciences ( Descritores em Ciências da Saúde, DeCS) for LILACS; Embase thesaurus (EMTREE) for Embase and CINAHL subjects for the CINAHL database; synonyms of the controlled descriptors of the databases themselves were also used, called “entry terms” in MeSH and “synonyms” in DeCS. After defining the search terms, they were associated with the AND and OR Boolean operators. 

Initially, the search strategy was applied with the MeSH terms and later on translated to the other terms. The search strategy with MeSH terms used was as follows: (“Hypertension”[MeSH] OR (Hypertension) OR (Blood Pressure, High) OR “Essential Hypertension”[MeSH] OR (Essential Hypertension) OR (Hypertension, Essential)) AND (“Nursing”[MeSH] OR (Nursing) OR “Cardiovascular Nursing”[MeSH] OR (Cardiovascular Nursing) OR (Cardiac Care Nursing) OR (Cardiac Nursing) OR (Cardiac Vascular Nursing) OR (Vascular Nursing) OR “Primary Care Nursing”[MeSH] OR (Primary Care Nursing)) AND (“Office Nursing”[MeSH] OR (Office Nursing) OR (Nursing, Office) OR “Control Groups”[MeSH] OR (Control Groups)) AND (“Arterial Pressure”[MeSH] OR (Arterial Pressure) OR (Arterial Blood Pressure) OR (Arterial Tension) OR (Blood Pressure, Arterial) OR (Mean Arterial Pressure) OR “Treatment Adherence and Compliance”[MeSH] OR (Treatment Adherence and Compliance) OR (therapeutic adherence) OR (treatment adherence)) AND (“clinical trial”[MeSH] OR (clinical trial) OR (intervention study)).

### Search selection

The results from each database were e-imported into the EndNote Basic reference manager, online version, to later remove the duplicates. The titles and abstracts of the publications were examined independently by two researchers. Subsequently, both reviewers dually and independently evaluated the full texts of the potentially eligible studies regarding compliance with the eligibility criteria. Any and all disagreements were solved by means of a discussion between the reviewers or with the participation of a third reviewer.

### Data extraction

Data extraction from the studies selected for the final sample was in charge of two independent researchers for a subsequent comparison. There were no divergences in the consensus meeting. A clinical form prepared by the researchers was used, containing the following: characteristics of the study (title, authors, year, country, objective); of the sample in the intervention and control groups (gender, age, size, recruitment locus), of the intervention (description, frequency, duration in weeks), and of the result (outcome, main results), and conclusion. The results were synthesized in a Microsoft Excel ^®^ spreadsheet. The Preferred Reporting Items for Systematic Reviews and Meta-Analyses (PRISMA) flowchart was used to document selection of the studies. 

### Bias assessment

The Revised Cochrane risk-of-bias tool for randomized trials (RoB 2) was adopted to analyze the risk of bias of the primary studies. The following domains were evaluated for internal validation: bias resulting from the randomization process; bias due to deviations from the intended interventions; bias due to missing result data; bias in measurement of the results; and bias in selection of the reported result. Finally, as a result of the risk assessment, each study was categorized as follows: low risk of bias, uncertain risk of bias or high risk of bias ^( 10)^ . Each stage was in charge of two independent reviewers and, in case any disagreement arose, they were solved by means of a discussion between the reviewers or with the participation of a third reviewer. The RoB 2 tool, version 22 of August 2019, was used to synthesize the risk of bias analysis of the studies. 

### Synthesis of the results

A qualitative synthesis was performed with a description of the characteristics of the studies, as well as a quantitative synthesis with the meta-analysis of the studies grouped. In the qualitative synthesis, for the classification of arterial hypertension, the values that classify the blood pressure behavior in adults that are included in the Brazilian Arterial Hypertension Guidelines were used ^( 2)^ . 

In the quantitative synthesis, in the case of missing data in the individual studies, even after contact with the authors of each primary study, data imputation was performed, when possible, according to the guidelines set forth in Chapter 6 of the Cochrane Handbook Manual. The meta-analyses were calculated using the Review Manager program, version 5.4.1 (RevMan 5.4.1), by the Cochrane Collaboration, presented through the Forest Plot graph and in the subgroups assembled for the meta-analysis.

Heterogeneity between studies was statistically evaluated using Pearson’s chi-square test (χ²) for its significance and the I-square (I²) inconsistency test for its magnitude. In relation to χ², a more conservative significance level (p<0.10) was considered to evidence the presence of heterogeneity. For the I² value, the scale rating according to Cochrane was considered ^( 10)^ : from 0% to 40%: heterogeneity may not be important; from 30% to 60%: it can represent moderate heterogeneity; from 50% to 90%: it can represent substantial heterogeneity; and from 75% to 100%: considerable heterogeneity. 

Regarding the statistical model chosen for the meta-analysis, it was determined by exploring heterogeneity, according to Cochran’s Q test and to the I² statistical value. The random effect model was chosen when heterogeneity was between substantial and considerable, and fixed effect when it was moderate or unimportant ^( 11)^ . As an effect measure, the continuous variable of the SBP and DBP values was used. For the means difference (MD) calculation a 95% confidence interval (CI) was considered, where an MD value below zero means a favorable outcome for the intervention. The overall effect of the intervention was considered statistically significant when p-values<0.05 were identified. 

### Certainty of the evidence assessment

To classify certainty of the evidence, the Grading of Recommendations Assessment, Development and Evaluation (GRADE) system from Cochrane ^( 12)^ was used. The criteria evaluated were methodological limitations (risk of bias), inconsistency, indirect evidence, imprecision, and publication bias. Certainty of the evidence was classified as follows: high, moderate, low or very low. In this process, the online GRADE Working Group tool was used, which can be accessed free of charge at [http://www.gradeworkinggroup.org]. 

## Results

### Characteristics of the studies included

Once the searches in the databases were concluded, 1,692 publications had been identified. A total of 1,265 remained after removing duplicates. After reading the titles and abstracts, 60 publications were listed as potentially relevant. After reading the articles in full, 52 publications were excluded for not meeting the eligibility criteria, with eight studies remaining ( Figure 1). The reasons for exclusion at this stage were as follows: studies with preliminary data, not presenting systolic or diastolic blood pressure as outcome; patient with chronic disease or hospitalized; intervention performed by a multidisciplinary team; or not having any Nursing intervention, in-person educational intervention combined with another form of intervention; clinical trial protocol; and study with data derived from another publication. 

In Figure 2, the general characteristics of the Randomized Clinical Trials (RCTs) that were selected for the systematic review are verified, with the following variables: author’s name, year of publication of the article, country where the research was carried out, characteristics of the sample, data from the educational intervention carried out by nurses, outcomes considered in the research, and main results. 

**Figure:**
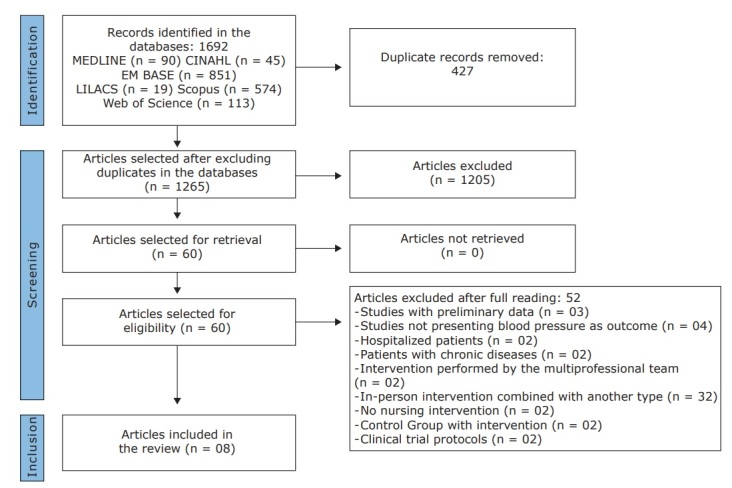
Figure 1 – Flowchart corresponding to selection of the articles according to the PRISMA recommendations. 2020

**Figure 2:** Characteristics of the randomized clinical trials included in the review (n=8). Teresina, PI, Brazil, 2022

Author / Year / Country	Characteristics of the sample	Educational intervention performed by nurses	Outcomes	Main results
Bogner, et al., 2013. United States ^( 13)^	n= 60 (GI ^*^/ GC † = 30/30) Age: 67.1±11.0 years-old Female: 65.0% IG^*^ = PA‡ media = 133,6/76,5 mmHg	Type: Individual in medical office Length of the intervention in time: 3 months / Monitoring time: 3 months	SBP § DBP || Depression	In relation to the CG †, the SBP § and DBP || values in the IG * were reduced by -8.10 mmHg (p=0.079) and -7.50 mmHg (p=0.035), respectively.
Bolarinwa, et al., 2019. Nigeria ^( 14)^	n = 299 (IG */CG † = 149/150) Age: 61.1±10.8 years old Female: 77.2% IG ^*^ = Mean BP ‡ = 139.3/86.5 mmHg	Type: Individual during home visit Length of the intervention in time: 6 months / Monitoring time: 6, 12 months	SBP § DBP || Adherence Quality of Life	In relation to the CG †, the SBP § and DBP || values in the IG * were reduced by -6.97 mmHg (p=0.013) and -4.08 mmHg (p=0.014), respectively.
Colósimo, et al., 2012. Brazil ^( 15)^	n = 82 (IG */CG † = 42/40) Age: 60.0±10.8 years old Female: 56.1% IG * = Mean BP ‡ = 135.0/78.7 mmHg	Type: Individual in medical office Length of the intervention in time: 6 months / Monitoring time: 6 months	SBP § DBP ||	In relation to the CG †, the SBP § and DBP || values in the IG * were reduced by -8.30 and -5.00 mmHg , respectively.
Drevenhorn, et al., 2012. Sweden ^( 16)^	n = 213 (IG */CG † = 153/60) Age: NR ¶ Female: NR ¶ IG * = Mean BP ‡ = 159.1/93.0 mmHg	Type: Individual in medical office Length of the intervention in time: 24 months / Monitoring time: 24 months	SBP § DBP || Lipid	In relation to the CG †, the SBP § and DBP || values in the IG * differed by -2.70 and +0.50 mmHg, respectively.
Khadoura, et al., 2021. Palestine( 17)	n = 355 (IG */CG † = 182/173) Age: 55.7±10.7 years old Female: 63.3% IG * = Mean BP ‡ = 133.3/85.3 mmHg	Type: Individual combined with group activity Length of the intervention in time: 3 months / Monitoring time: 4 months	SBP § DBP || Adherence	In relation to the CG †, the SBP § and DBP || values in the IG * were reduced by -3.70 and -3.30 mmHg , respectively.
Kolcu, et al., 2020. Turkey ^( 18)^	n = 76 (IG */CG †= 38/38) Age: 75.6±7.2 years old Female: 48.6% IG * = Mean BP ‡ = 129.1/79.7 mmHg	Type: Individual combined with group activity Length of the intervention in time: 5 months / Monitoring time: 6 months	SBP § DBP || Lipid Adherence	In relation to the CG †, the SBP § and DBP || values in the IG * were reduced by -4.44 mmHg (p=0.000) and -3.01 mmHg (p=0.003), respectively.
Ma, et al., 2014. China ^( 19)^	n = 120 (IG */CG †= 60/60) Age: 58.7±11.6 years old Female: 50.8% >IG * = Mean BP ‡ = 153.1/89.0 mmHg	Type: Individual in medical office Length of the intervention in time: 6 months / Monitoring time: 6 months	SBP § DBP || Adherence Quality of Life	In relation to the CG †, the SBP § and DBP || values in the IG * were reduced by -4.92 mmHg (p=0.011) and -2.58 mmHg (p=0.027), respectively.
Shamsi, et al., 2021. Iran ^( 20)^	n = 50 (IG */CG †= 25/25) Age: 56.9±7.5 years old Female: 42.0% IG * = Mean BP ‡ = 144.2/89.0 mmHg	Type: Individual combined with group activity Length of the intervention in time: 4 months / Monitoring time: 4 months	SBP § DBP || Sodium intake	In relation to the CG †, the SBP § and DBP ^||^ values in the IG * were reduced by -13.20 mmHg (p=0.001) and -7.00 mmHg (p=0.011), respectively.

* IG = Intervention Group;

† CG = Control Group;

‡ BP = Blood Pressure;

§ SBP = Systolic Blood Pressure;

|| DBP = Diastolic Blood Pressure;

¶ NR = Not Reported

All eight RCTs selected for this systematic review accounted for a total of 1,255 participants and the mean age of the samples in the studies was over 55 years old, with prevalence of aged individuals. The research studies were conducted in the following countries: United States ^( 13)^ , Nigeria ^( 14)^ , Brazil ^( 15)^ , Sweden ^( 16)^ , Palestine ^( 17)^ , Turkey ^( 18)^ , China ^( 19)^ and Iran ^( 20)^ ; and they were published between 2012 and 2021. 

As for gender, five of the studies (62.5%) had more females in their samples ^(13-15,17,19)^; the male gender was prevalent in two studies (25.0%) ^(19-20)^ ; and one of the studies (12.5%) did not report the characteristics regarding the research participants’ gender and age ^( 16)^ . Among these publications, the study conducted in Brazil ^( 15)^ presented a statistically significant difference regarding gender between the intervention and control groups at baseline. 

As for the way in which the in-person educational intervention was carried out by the nurses, it was identified that it was both individually, in the office ^( 13, 15- 16, 19)^ or during home visits ^( 14)^ , and combined with a group activity ^( 17- 18, 20)^ . As for the duration of the educational intervention carried out by nurses, it was verified that it varied from 3 months to 24 months, with two studies (25.0%) where it lasted up to 3 months ^( 13,17)^ , from 4 to 6 months in five studies (62.5%) ^( 14- 15, 18- 20)^ and 24 months in one study (12.5%) ^( 16)^ . 

In relation to the time between completion of the intervention and post-intervention data collection time, it was identified that five studies (62.5%) carried out data collection immediately after finishing the intervention ^( 13, 15- 16, 19- 20)^ , two studies (25.0%) had a 1-month time interval ^( 17- 18)^ and one study (12.5%) had a 6-month interval ^( 14)^ after completing the intervention. 

As for the SBP and DBP outcomes, four studies (50.0%) reported how the blood pressure checks were performed ^( 15, 17- 19)^ , one study (12.5%) reported that it followed the American Heart Association Guidelines ^( 13)^ , one study (12.5%) asserted following a standard protocol of the institution where patients undergo treatment ^( 20)^ , and another two studies (25.0%) did not report how the SBP and DBP measurements were performed, only informing the respective values ^( 14, 16)^ . Other outcomes were also identified in the studies, such as the following: adherence, quality of life, Body Mass Index, lipid values, sodium intake, smoking habit and depression. 

In the SBP outcome, all studies (100.0%) obtained, in the post-intervention verification, lower blood pressure figures in the Intervention Group when compared to those of the Control Group ^( 13- 20)^ , with a reduction between groups varying from -2.70 mmHg to -13.20 mmHg. However, only four studies (50.0%) confirmed a statistically significant difference in SBP reduction between the groups ^( 14, 18- 20)^ . 

In the DBP outcome, only the study conducted in Sweden ^( 16)^ did not present any reduction in such value when comparing the intervention and control groups after the intervention. However, when the SDP reduction is verified, in each of the study groups it was evidenced that the reduction in the Intervention Group (-9.4 mmHg) was higher than in the Control Group (-7.1 mmHg). Five studies (62.5%) confirmed a statistically significant difference in DBP reduction between the groups ^( 13- 14, 18- 20)^ . 

### Risk of bias assessment

Figure 3 presents the result of the risk of bias assessments corresponding to each of the RCTs included in this review, by using the RoB 2 tool. 

**Figure:**
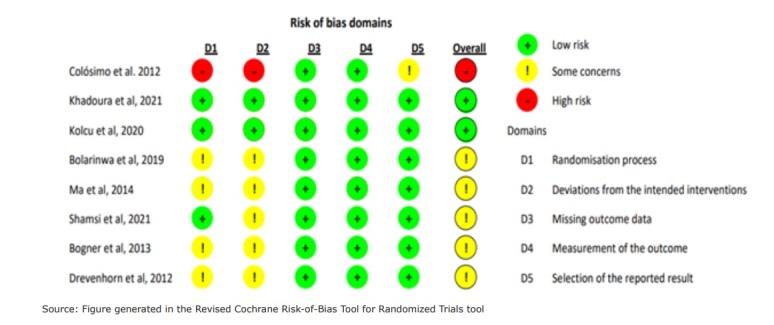
Figure 3 - Risk of bias assessments corresponding to the randomized clinical trials in each of the RoB 2 domains. Teresina, PI, Brazil, 2022

Source: Figure generated in the Revised Cochrane Risk-of-Bias Tool for Randomized Trials tool

The studies carried out in Palestine and Turkey ^( 17- 18)^ were assessed as with low risk of bias; those performed in the United States, Nigeria, Sweden, China and Iran ^( 13- 14, 16, 19- 20)^ as with uncertain risk of bias; and the one conducted in Brazil ^( 15)^ as with high risk of bias. The study classified as with high risk of bias was due to concerns about the randomization process for not having concealed the allocation sequence and presenting baseline differences between the groups (Domain 1) and referring to the deviation from the intended interventions for not reporting in the study whether there was non-adherence to the intervention that might have affected the outcome and whether an appropriate analysis was used to estimate the adherence effect (Domain 2). 

### Meta-analysis

**Figure 4:**
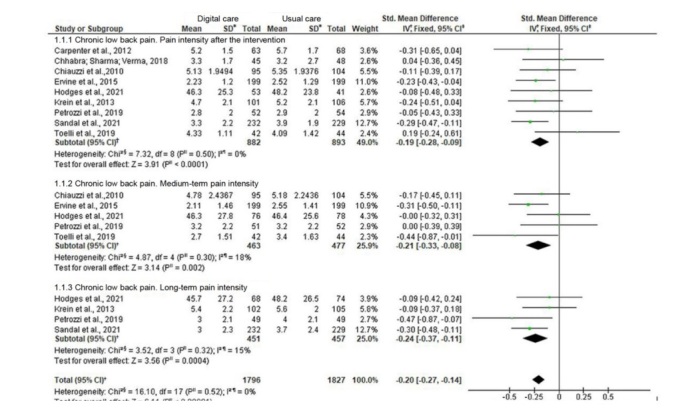
Meta-analysis of the effect of the in-person educational intervention performed by nurses versus usual care for the “systolic blood pressure” and “diastolic blood pressure” outcomes. Teresina, PI, Brazil, 2022

Source: Figure generated in the RevMan® statistical program; SD = Standard Deviation; IV = Interval of Variance; CI = Confidence Interval

The eight RCTs selected for this review were included in the meta-analysis, creating subgroups for the PAS and PAD outcomes, as well as subgroups by intervention longer than 3 months and by way of carrying out the in-person educational intervention. Figure 4 4presents the Forest Plot for each subgroup of the in- person educational intervention performed by nurses on people with arterial hypertension, when compared to usual care. 

In this meta-analysis, with eight studies, the in-person educational intervention performed by nurses on people with arterial hypertension is contemplated, regardless of the time the intervention was carried out, showing a -6.85 mmHg reduction in SBP (95% CI: from -9.65 to -4.05, p<0.00001, I ^2^=54%) and a -3.78 mmHg reduction in DBP (95% CI: from -5.00 to -2.55, p<0.00001, I ^2^=7%). In the subgroup, considering the in-person educational intervention carried out by nurses, individually, in the office and intervention longer than 3 months, it was possible to align three studies ^( 15- 16, 19)^ , obtaining a -3.78 mmHg reduction (95% CI: from -6.71 to -0.85, p=0.01, I ^2^ = 0%, n=840 participants) for the SBP outcome and a -2.30 mmHg reduction (CI 95%: from -5.60 to 1.00, p=0.17, I ^2^=30%, n=840 participants) for the DBP outcome. 

**Figure 5:**
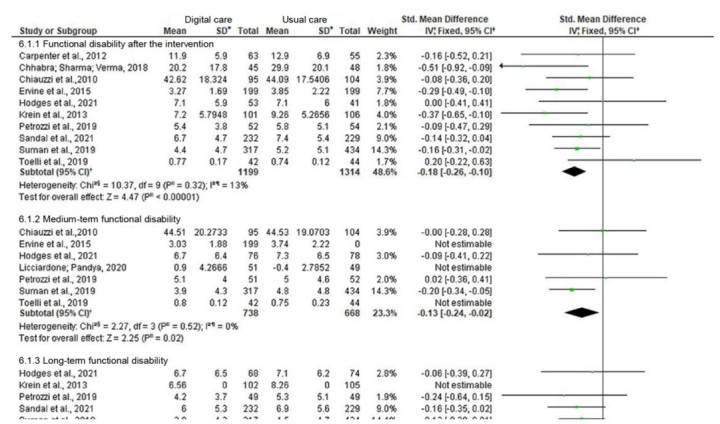
Meta-analysis of the effect of the in-person educational intervention performed by nurses, individually and combined with a group activity, versus usual care for the “systolic blood pressure” and “diastolic blood pressure” outcomes. Teresina, PI, Brazil, 2022

Source: Figure generated in the RevMan® statistical program; SD = Standard Deviation; IV = Interval of Variance; CI = Confidence Interval

Figure 5 presents the Forest plot for each subgroup of the in-person educational intervention performed by nurses, individually and combined with a group activity, when compared to usual care.

In the subgroup, considering the in-person educational intervention performed by nurses, individually, in the office combined with the group activity and interventions lasting more than 3 months, two studies were grouped ^( 18, 20)^ achieving a -12.41 mmHg (95% CI: from -16.91 to -7.91, p<0.00001, I ^2^=0%) reduction in SBP and a -5.40 mmHg (95% CI: from -7.98 to -2.82, p<0.0001, I ^2^=0%) reduction in DBP. 

### Certainty of the evidence assessment

Certainty of the evidence was assessed according to the SBP and DBP outcomes for the types of interventions performed by nurses, resorting to GRADE ^( 12)^ . For the meta-analysis, in the comparison between the in-person educational intervention and usual care to reduce SBP, quality of the evidence was considered low (confidence in the effect estimate is limited) for presenting the risk of bias classified as high in the evaluation of the study carried out in Brazil ^( 15)^ , in addition to presenting a serious inconsistency, that is, substantial heterogeneity (I ^2^=54%). For the DBP outcome, quality of the evidence was considered moderate (moderate confidence in the estimated effect), also due to the high risk of bias rating in the same study. 

In the meta-analysis of the comparison of in-person educational intervention performed by nurses, individually and in the office combined with group activity versus usual care, quality of the evidence, both for the “reduction in the SBP value” and “reduction in the DBP value” outcomes and according to GRADE ^( 12)^ , was classified as high (there is strong confidence that the true effect is close to the estimated one). 

## Discussion

In this systematic review with meta-analysis, different forms of educational interventions carried out by nurses, in person, that contribute to blood pressure control in people with hypertension were identified. However, even though there was heterogeneity across the studies included regarding length of the intervention in time, it was possible to obtain a synthesis of the best scientific evidence on the theme, which contributes to notoriety and use of these Nursing interventions in the clinical practice.

It is evident that arterial hypertension is a serious health problem in Brazil and in the world, becoming a major challenge for global health. Thus, it is indispensable that health professionals, including nurses, resort to interventions with scientific evidence to carry out clinical care that seeks to contribute to reducing blood pressure values. The essence of clinical care nurses provides intersubjective meeting spaces between professionals and people who experience a chronic health condition, necessary for the development of attitudes and changes in behavior. In this way, the nurses’ role has great potential to act according to the chronic care assumptions, whether in Nursing consultations or in individual or collective educational activities, even in mobilization actions in the community ^( 21)^ . 

In this perspective, performance of the in-person educational intervention by nurses in all eight RCTs of this review was analyzed and classified in three different ways with regard to its execution: individually, only nurse and patient, performed in the office ^( 13, 15- 16, 19)^ , individually during the home visit ^( 14)^ , and individually in the Nursing office combined with group activity meetings carried out by nurses ^( 17- 18, 20)^ . 

The study conducted in Nigeria ^( 14)^ had the intervention performed individually during home visits that took place monthly, although the duration of each home visit was not specified. During the face-to-face moments, there was a health education session, counseling on AH, and monitoring of blood pressure and Body Mass Index. In this intervention, SBP and DBP were checked immediately after the end of the intervention and after 6 months, with mean SBP values of 132.97±20.49 mmHg and 135.89±20.92 mmHg, respectively. It was verified that, after 6 months without performing the intervention, there is an increase in the SBP value. The same happened in relation to the DBP value: it presented a mean of 82.66±11.63 mmHg immediately at the end of the intervention and a mean of 84.69±12.83 mmHg 6 months after the end of the intervention, also verifying an increase in the values after ceasing the intervention. 

In the cases in which the intervention took place individually in the office, two studies ^( 13, 15)^ started, at baseline, with their samples presenting blood pressure classified as pre-hypertension and, in the other two studies ^( 16, 19)^ , blood pressure was classified as Stage 1 hypertension. As for the time interval between one in-person consultation and another, it varied from 2 to 6 weeks and each consultation lasted a mean of 30-40 minutes in two studies ^( 13, 19)^ , whereas the other two studies did not report this information ^( 15- 16)^ . 

The subgroup of the in-person educational intervention performed by nurses individually, without considering the intervention length in time ^( 13, 15- 16, 19)^ , presented a mean reduction effect estimate of -4.82 mmHg in SBP (95% CI: from -8.10 to -1.54, p=0.004, I ^2^=14%) and of -3.15 mmHg (95% CI, -6.46 to 0.16, p=0.06, I ^2^=36%) in DBP. When considering the intervention length in time equal to or greater than 3 months, three studies ^( 15- 16, 19)^ were included in the subgroup that presented -3.78 mmHg (95% CI: from -6.71 to -0.85, p=0.01, I ^2^=0%) for SBP and -2.30 mmHg (95% CI: -5.60 to 1.00, p=0.17, I ^2^=30%) for DBP. 

It can be seen that, regardless of time, the intervention has a statistically significant value for the SBP reduction; however, for the effect on DBP, it is not possible to reject the null hypothesis because the diamond crosses the nullity line. Analyzing the reduction in the blood pressure values between the intervention groups of the studies that had blood pressure classified as Stage 1 arterial hypertension at the baseline, it was identified that the SBP and DBP reduction values in each study group, comparing the baseline and post-intervention values, presented clinically significant reductions, with mean values of -11.36 mmHg in SBP and -6.46 mmHg in DBP in the study conducted in China ^( 19)^ and mean drops of -16.2 mmHg in SBP and -9.4 mmHg in DBP in the study conducted in Sweden ^( 16)^ . 

As for the in-person educational intervention performed by nurses, combining individual meetings with group activity moments, the studies were carried out in Palestine ^( 17)^ , Turkey ^( 18)^ and Iran ^( 20)^ . Performing an individual analysis of each study of this intervention, it can be reported that the study carried out in Palestine had its sample at baseline with blood pressure classified as pre-hypertension and that the intervention lasted 3 months, with only a single individual moment lasting 30 minutes, combined with two group moments, with a mean duration of 20-30 minutes each meeting. In this study, when comparing the decrease in the blood pressure levels between the baseline and the post-intervention, the mean reductions were -4.3 mmHg and -3.2 mmHg for SBP and DBP, respectively. 

The study conducted in Turkey ^( 18)^ had its sample at baseline with blood pressure classified as normal and the intervention lasted 5 months, with 6 Nursing consultations carried out weekly, combined with four moments in group, held weekly and interspersed with Nursing consultations. The duration of the individual meetings or group activities was not informed. In this study, the Intervention Group had mean reductions of -10.5 mmHg in SBP and -1.89 mmHg in DBP, respectively. The study conducted in Iran ^( 20)^ had its baseline sample with blood pressure classified as Stage 1 hypertension and the intervention lasted 4 months, with 12 Nursing consultations carried out weekly and a mean duration of 40 minutes each, combined with 8 group moments, 2 meetings per week with a mean duration 

of 60 minutes each group activity. In the Intervention Group of this study, 15.8 mmHg and -10.5 mmHg reductions were identified in SBP and DBP, respectively.

Performing individual analysis of each intervention group, it can be asserted that the more face-to-face moments offered to the patient to carry out health education, the greater the reduction in the blood pressure values. When a meta-analysis of this intervention was performed ^( 17- 18, 20)^ , a mean reduction of -9.04 mmHg was obtained for the SBP outcome (95% CI: from -15.84 to -2.25, p=0.009, I ^2^=81%, n=481 participants) and, for the DBP outcome, the mean reduction was -4.04 mmHg (95% CI: from -5.68 to -2.41, p<0.00001, I ^2^=15%, n=481 participants). In this subgroup, a statistically significant reduction both in SBP and in DBP is confirmed, although heterogeneity classified as substantial was identified in the SBP outcome. 

Due to the high statistical heterogeneity presented in the meta-analysis of the studies with the educational intervention performed individually combined with group activity for the SBP outcome, a subgroup of this intervention was carried out considering the intervention time equal to or greater than 3 months ^( 18, 20)^ . In this subgroup, heterogeneity was classified as unimportant (I ^2^=0%) and obtained a mean reduction of -12.41 mmHg in SBP (95% CI: from -16.91 to -7.91, n=126 participants) and of -5.40 mmHg in DBP (95% CI: from -7.98 to -2.82, n=126 participants). When evaluating quality of the evidence, according to GRADE, this subgroup considering time, the assessment of the evidence was classified as high both for the SBP and DBP outcomes. 

The meta-analysis of the subgroup of the in-person educational intervention performed by nurses, individually combined with group activity, lasting at least 3 months, shows a statistically significant reduction in SBP and DBP, as well as, according to a meta-analysis carried out in 2016 ^( 8)^ , it proves a clinically significant effect, thus confident for the reduction in the risk of major cardiovascular events. 

As limitations found in this research, we mention that few studies met the inclusion criteria for this systematic review, especially studies carried out in Brazil. In addition, some primary studies had small samples, both in the intervention and in the control group. One of the RCTs included in this review presented high risk of bias in two of the five domains of the RoB 2 assessment. In addition to that, the absence of detailed information on the description of the intervention performed by nurses is highlighted, such as the number of interventions and the duration of each face-to-face moment between the nurse and the hypertensive patient.

In terms of future studies, it is recommended that future RCTs have larger samples so that the effect of the intervention performed by nurses can be better evaluated. It is also suggested that there should be a more refined description of the intervention performed by nurses, as well as the usual care performed on the sample, so that a comparison with other studies can be carried out in terms of control with usual care.

It is also important to reinforce the need for clinical trial studies, with this intervention and of good methodological quality, to be carried out in Brazil, as this is the only way to be certain about the quality of the intervention in our country. It is also suggested that the cost of the intervention carried out by nurses be evaluated, as it is relevant to consider whether this intervention is financially beneficial or not to this population group. It is believed that implementing interventions with a preventive approach can stand out in relation to the high financial costs resulting from hospitalizations associated with arterial hypertension in the SUS.

## Conclusion

It was evidenced that the in-person educational intervention performed by nurses, especially when carried out individually, through the Nursing consultation and combined with group activities, has a considerable clinical impact on reducing the SBP and DBP values in people with arterial hypertension, being extremely useful to controlling the blood pressure levels and, consequently, to reducing comorbidities, hospitalizations and premature deaths due to cardiovascular diseases.

It is expected that the findings of this systematic review with meta-analysis will encourage managers and public health services to provide resources so that nurses can carry out the intervention, as well as sensitize nurses to recognize and apply in Primary Health Care the interventions that have shown proven benefits in reducing systolic and diastolic blood pressure in people with high arterial hypertension.
